# Characterization of nitroreductase gene A of *Enterococcus faecalis* isolates from fecal samples of patients with colorectal cancer and healthy people

**DOI:** 10.1515/biol-2025-1276

**Published:** 2026-05-18

**Authors:** Ping Li, Xiang Li, Jianing Li, Yuqi Tan, Mingcheng Li

**Affiliations:** Department of Clinical Laboratory, Chemical Industry’s Hospital of Jilin, Jilin, China; Department of Colorectal Surgery, People’s Hospital of Jilin, Jilin, China; Department of Clinical Microbiology, School of Medical Technology, Beihua University, Jilin 132013, China

**Keywords:** *E. faecalis*, NfsA, colorectal cancer, activity differences

## Abstract

The mechanisms of *Enterococcus faecalis* (*E. faecalis*) in the oncogenesis of colorectal cancer (CRC) remain to be revealed. The study aims to identify oxygen-insensitive nitroreductase gene A (NfsA) in *E. faecalis* between patients with CRC and healthy subjects. A total of 51 patients with CRC were enrolled as an observational group and 30 healthy controls were included in this study during July 2022 to July 2024. *E. faecalis* isolates were selectively identified from stool samples from two groups. The NfsA genes were analyzed using cloning and sequencing techniques. The fluorescence quantitative PCR targeting the NfsA gene was employed to explore difference of gene expression levels. The differences of the enzymatic activity were also evaluated. A total of 81 isolates were selectively isolated and identified as *E. faecalis* from stool samples. The exact point mutation exhibited that the base mutated from GA to AC at 52 and 53rd, G to A at 67th, G to A at 105th, C to T at 506th, A to C at 509th in addition to a base deletation at 408, 409th. The mutations frequencies showed statistically significant (*P* < 0.05). The mRNA expression of the NfsA and the enzymatic activity in fecal samples of patients with CRC was significantly higher than that in healthy controls (*P* < 0 05). The mutation, expression, and activity of NfsA in *E. faecalis* from feces of patients with CRC are closely related to the occurrence and development of CRC. Our findings showed that *E. faecalis* colonizing within intestine may play a role in the tumorigenesis.

## 1 Introduction

Colorectal cancer (CRC) is one of the most common malignancies worldwide, being the fourth most common cause of cancer-related mortality [1]. Although progress has been made in prevention and treatment, it is still the second deadliest tumor after lung cancer and the incidence of CRC continues to increase among adolescents and young adults (AYA) patients, who are defined as individuals under 45 years of age [2], 3]. Among them, dietary habits, family heredity, and living environment are important conditions related to the occurrence of CRC [4], 5]. The occurrence and development of intestinal flora and intestinal cancer is the key focus of much research at time of writing. Many researchers have focused on gastrointestinal microflora, a complex symbiotic flora composed of 10^14^ individual bacteria and more than 3 million genes [6]. The gut microbiota plays a crucial role in food digestion, immune activation, and regulation of gut endocrine signaling pathways. Meanwhile, they closely communicate with the central nervous system (CNS) and other parts of the body by producing specific metabolic compounds [7]. In recent years, a large number of studies have indicated that gut microbiota is closely related to the occurrence of CRC. Metagenomics and transcriptional reporters have revealed that the expressions of specific bateria, including *Enterococcus faecalis* (*E. faecalis*) in addition to *Fusobacterium nucleatum* in human CRC tissues are significantly increased [8], 9], compared with adjacent normal tissues. Whether the high enrichment of *E. faecalis* is a reason for CRC or it is a result of CRC? A number of studies have been conducted on *E. faecalis* to clarify its role in the occurrence and development of CRC [8], 10].

Nitroreductases (NfsAs) have been isolated from a large number of bacterial species in enteric bacteria, which encode a minor oxygen-insensitive enzyme that catalyzes reduces nitroaromatic compounds, and are considered for biodegradation of nitroaromatic pollutants [11]. *E. faecalis* is a Gram-positive, facultative anaerobic bacterium that is a common bacterium in human intestinal microorganisms, which plays an important role in maintaining intestinal homeostasis [9], 12]. NfsA in *E. faecalis* can reduce nitrate in the human gastrointestinal tract to active and toxic nitrite. Nitrite is further combined with nitrogen-containing compounds to produce nitroso compounds, which are highly carcinogenic [13]. Recent studies have shown that nitrogen-containing compounds are associated with changes in the human gene expression, demonstrating the potential role of these compounds in the development and progression of CRC [11], 14]. The carcinogenic role of *E. faecalis* in CRC was found to be related to its ability produce reactive oxygen species (ROS) and superoxide (SOD) that brought about genomic instability, damaged colonic DNA and caused gene mutation and cancer in the host [13], 15]. Moreover, NfsAs are one of the most candidates for gene-directed enzyme-prodrug therapy in anti-cancer strategy [16]. The study aimed to compare the characteristics of NfsA sequence, mRNA expression, and enzymatic activity in *E. faecalis* isolates from fecal samples of patients with CRC and healthy people, to confirm the NfsA activity and enzymes possibly involved in CRC.

## 2 Materials and methods

### 2.1 Patient selection

A total of 51 patients with CRC treated in Cancer Centre, Chemical Industry’s Hospital of Jilin were enrolled as an observational group in this study during July 2022 to July 2024. Inclusion of patients with CRC in this study: ① the patients whose clinical symptoms coincided with the diagnostic criteria for CRC, and the patients who were diagnosed with CRC by pathological and serological examination; ② No digestive tract diseases (such as pancreatitis, intestinal obstruction, etc.); ➂ Patients who have provided informed consent to participation in the present study; Exclusion criteria: ① Patients who received adjuvant treatment such as operation, radiotherapy, or chemotherapy for CRC; ② Excluding those with severe impairment of body function (such as impairment of heart, lung, liver, or other organ functions; ➂Exclusion of familial hereditary digestive tract diseases (such as colorectal adenomas, intestinal inflammatory diseases, etc.); ④ Exclusion of patients with possible distribution and metabolic disorders of intestinal flora in the past month (such as antibiotics, microecological agents, etc.). Meanwhile, 30 healthy controls were included from the Physical Examination Centre, Chemical Industry’s Hospital of Jilin. Inclusion of healthy controls: ① Healthy controls without familial or chronic intestinal diseases; ② Healthy controls whose physical examination indexes were within the normal range; ➂ The distribution and metabolism of intestinal flora are normal and remained thus in the near future; ④ Patients who have provided informed consent to participation in the present study.

**Informed consent:** Informed consent has been obtained from all individuals included in this study.

**Ethical approval:** The research related to human use has been complied with all the relevant national regulations, institutional policies and in accordance with the tenets of the Helsinki Declaration, and has been approved by the Institutional Ethics Committees of the participating Hospitals (Protocol Number: JLHG-IEC-2023-019-01).

### 2.2 Isolation, culture, and identification of *E. faecalis isolates*

About 2 g the fresh rectal stumps or 2 ml rectal fluids through endoscope were collected from patients with CRC and healthy controls. They were transported to the laboratory at 4 °C and were kept refrigerated and processed within 4 h. Faecal samples were added to 5 ml of peptone water, being oscillated at 37 °C for 2 h, the dilutions were inoculated in Pfizer Enterococcus Selective Agar (PSE) and incubated at 37 °C for 24 h. Colonies of suspected *E. faecalis* were picked out and smears were Gram-stained to observe the bacterial morphology. Simultaneously, the cultivated colonies were inoculated into the V-K 2 Compact identification card (VITEK, bioMérieux, Marcy-l’Étoile, France). Finally, the determinged colonies were picked with a cotton swab, dried at room temperature, and 70 % formic acid (1 μL) was added. After drying at room temperature, 1 μL of the substrate solution was added and dried, then placed the target plate on the MALDI-TOF mass spectrometer (bioMe´rieux, Marcy I’Etoile, France) for identification and recorded the results. *Escherichia coli* (ATCC7893) and *E. faecalis* ATCC 29212 were used as the control strains.

### 2.3 Extraction and analysis of genomic DNA

A bacterial genome extraction kit (Omega, USA) was used to extract the genome from *E. faecalis* according to the company construction. The bacterial genome extracts were dissolved in sterilized water, and stored at −20 °C for further analysis. The DNA concentration was calculated using an ultraviolet spectrophotometer (Q6000, Quawell, USA). The purity of each sample was determined according to the *A*_260_/*A*_280_ ratio. Meanwhile, 5 μL of DNA extracts were subjected to electrophoresis on a nucleic acid dye containing 0.8 % agarose gel (GelRed 0.5 mg/L). The result was examined using a UV gel imaging analyzer and photographed.

### 2.4 Primer design

The gene sequences for NfsA gene (No.WP_002360554.1) were downloaded from GenBank. Two specific primers for conventional PCR and fluorescence quantitative PCR were designed by NCBI Primer-BLAST online primer design software and synthesized by Sangon Biotech (Shanghai, China) Co., Ltd.

Primer 1: Forward primer, 5′-ATG​ACG​CCA​ACC​ATT​GAA​CTT​ATT​TGT​G-3′; Reverse primer: 5′-TTA​GCG​CGT​CGC​CCA​ACC-3′, for which the expected size was 723 bp.

Primer 2: Forward primer, 5′-GCG​GAG​TTC​TGG​GTG​TTC​TG-3′; Reverse primer, 5′-TGC​CGT​ATC​AAC​GAC​ACC​G-3′, which were applied in subsequent fluorescent quantitative PCR.

### 2.5 Amplicon, cloning, sequencing and blasting of the *NfsA* gene

The reaction system included 2 × Taq PCR Master Mix (TIGEN, BEIJING) 10 μL, forward primer and reverse primer (10 ng/μL) 1 μL, genomic DNA template (100 ng/μL) 1 μL, and sterilized, double-distilled water 7 μL, with a total system volume of 20 μL. Reaction procedure involved pre-denaturing at 95 °C for 5 min, denaturing at 95 °C for 30 s, annealing at 55 °C for 30 s, extending at 72 °C for 30 s and fully extending at 72 °C for 5 min. The PCR reaction was performed to amplify the full length of the NfsA gene. The 5 μL of PCR amplification products were added into a 2 % agarose gel electrophoresis plate that contained Gel Red. The voltage of the electrophoresis instrument was adjusted to 100 V, the electrophoresis instrument was turned off after 60 min of electrophoresis; finally, the UV analyzer (Thermo Scientific, USA) was used to observe the results.

The electrophoretic DNA fragments were purified and recovered by agarose gel purification kit (Takara Bio Inc., Japan), and the concentration and purity were measured. The NfsA gene was linked to the vector by pGM-T cloning kit (Takara Bio Inc., Japan), added to TOP10 competent cells, and 500 μL of preheated SOC culture solution (Promega, USA) was added to the culture bottle, and cultured at 37 °C for 3 h to resuscitate the cells. The mixed bacterial solution of 100 μL was absorbed and inoculated on the transformation plate and cultured overnight.

The white colonies on the plate were inoculated into 6 ml LB (containing 80 μg/ml Amp) liquid medium, and cultured overnight at 37 °C and 150 rpm. The plasmid in bacterial solution was extracted by plasmid extraction kit (Takara Bio Inc., Japan), and verified by PCR amplification and subsequent electrophoresis. The plasmid, which contained PCR amplification, was sent to Sangon Biotech (Shanghai, China) Co., Ltd for sequencing. Sequence similarity searching was conducted with the BLAST program available at the website of the NCBI (www.ncbi.nlm.nih.gov). Multiple sequence alignment was performed using Clustal X (1.8) to confirm the sequence comparison.

### 2.6 Extraction of total RNA from *E. faecalis* isolates and amplification of an internal reference gene

The total RNA from bacteria was extracted by TRI-ZOL kit (Takara Bio Inc., Japan), and the extracted total RNA of bacteria was reverse transcribed into cDNA using a reverse transcription kit (Takara Bio Inc., Japan). PCR amplification and agarose gel electrophoresis were conducted for verification. Taking cDNA as the target gene template, the cDNA of CRC patients and healthy controls were subjected to fluorescence quantitative PCR using a two-step method (under conditions entailing 40 cycle dissolution curves, pre denaturation at 95 °C for 10 min, denaturation at 95 °C for 15 s, annealing at 60 °C for 30 s, and extension at 72 °C for 1 min, which were automatically applied), and the unified threshold value was calculated. According to the standard curve, the copy number was finally converted into a concentration.

### 2.7 Detection of the *NfsA* mRNA expression by fluorescence quantitative PCR

SYBRGreen Ⅱ chimeric fluorescence method was used, and the reverse-transcribed cDNA sample was used as the template of real-time fluorescent quantitative PCR. The purified cDNA fragment (an internal reference gene from *NfsA*) was obtained in the previous step, and the copy number was obtained according to the formula used for conversion between concentration and copy number (Copy = *C* × N_A_ × 10^−9^ × 660 *A* × target gene base number, where N_A_ = 6.02 × 10^23^). The gradient was diluted to 10^9^,10^8^, 10^7^, 10^6^, 10^5^, 10^4^, and 10^3^ for fluorescence quantitative PCR (Applied Biosystems, Foster, CA, USA). The reaction procedure adopted for fluorescent quantitative PCR was as follows: pre denaturation at 94 °C for 10 min; denaturation at 95 °C for 15 s, annealing at 58 °C for 30 s, extension at 72 °C for 30 s, 40 cycles. The standard curve was automatically generated by the system according to the *C*_*t*_ value and copy number. This experiment selected ^2−ΔΔ^ *Ct* method using an internal reference gene to analyze and calculate the relative expression formula of the target gene as follows: ^2−ΔΔ^ *Ct* = 2 − [(*Ct* purpose − *Ct* internal parameter) Test − (*Ct* purpose − *Ct* internal parameter) Control].

### 2.8 Detection of the NfsA activity by an enzyme-substrate method

The specific substrate of NfsA, furacilin powder (Sigma, USA) was dissolved in PBS buffer solution to a prepared concentration of 0.1 mM, and was diluted to 0.1–0.01 mM in gradient steps. The absorbance was measured at 25 °C and 410 nm with an enzyme reader (Thermo Scientific, USA). According to the absorbance and its concentration, the labelling curve was plotted. A single colony was selected and inoculated with the single colony into a fermentation medium before centrifugation at 150 rpm, shaking for 10 h and culturing overnight; some 200 ml were absorbed and added to 96-well plates. The absorbance measured at a wavelength of 600 nm was 0.7. The bacterial culture solutions were then centrifuged at 4,000 rpm for 2 min and precipitates were suspended with PBS buffer. The specific substrate of NfsA and inducer ITPG (Sigma, USA) were dissolved in PBS buffer to prepare solutions with concentrations of 0.1 mM and 0.2 mM, respectively. The bacterial suspended solution (50 μL) and the 150 μL PBS buffer containing specific substrate and inducer were added to the 96-well plate (total volume was 200 μL). The solutions were then mixed into an incubator at 37 °C and cultured for 5 h. The absorbance was measured by an enzyme reader at 25 °C and a wavelength of 410 nm. The concentration was calculated according to the standard curve. According to the definition of the enzymatic activity, the NfsA activity of CRC patients and healthy controls was calculated according to formula *U*/*L* = (*C*_start_ − *C*_end_) × *V*/*h*. The C_start_ represented the initial concentration of substrate for NfsA and *C*_end_ represented the concentration stopding catalysis of substrate at a specific time. The V symbolised the volume and the h mean hour.

### 2.9 Statistical analysis

Three parallel groups were set for each sample, and the *Ct* values of internal reference gene and target gene were calculated using MS-Excel^®^ software. The relative mRNA level of each target gene was calculated by the *Ct* method. SPSS17.0 software was used for statistical processing and analysis. The experimental data were expressed by ®*X* ± *S* and *t*-test in the group; *P* < 0.05 was deemed to be indicative of a statistically significant difference.

## 3 Results

### 3.1 Demographic characteristics of all participants

The observational group included 31 male patients and 20 female patients with CRC aged between 43 and 77 years, and the average age was (61.33 ± 9.88) years. Whereas, the healthy controls included 17 males and 13 females, aged between 45 and 67, with an average age of (57.58 ± 5.79) years. Data analysis showed that there was no significant difference in general data such as gender distribution and age composition between two groups (*P < *0.05), except that fat-rich diet, there was a significant difference (*P* > 0.05) (Table 1).

**Table 1:** Demographic characteristics of all participants in the study [*n* (*η*/%)].

Variables	Observational group (*n* = 51)	Control group (*n* = 30)	*χ* ^2^	*P*-value
Age (mean ± SD, years)	61.33 ± 9.88	57.58 ± 5.79	0.04	>0.05^b^
Sex (male/female)	31/20	17/13	0.01	>0.05^b^
Fat-rich diet	19 (37.51 %)	6 (16.67 %)	18.34	<0.05^a^
Alcohol consumption history (>10 years)	10 (19.61 %)	7 (23.33 %)	3.23	>0.05^b^
Smoking history (>10 years)	9 (17.65 %)	4 (13.33 %)	9.14	>0.05^b^

^a^ *P < *0.05 indicates statistical significance; ^b^ *P* > 0.05 indicates no statistical significance compared to the control group determined by using the Student’s *t*-test.

### 3.2 Bacterial identification

**Figure 1:**
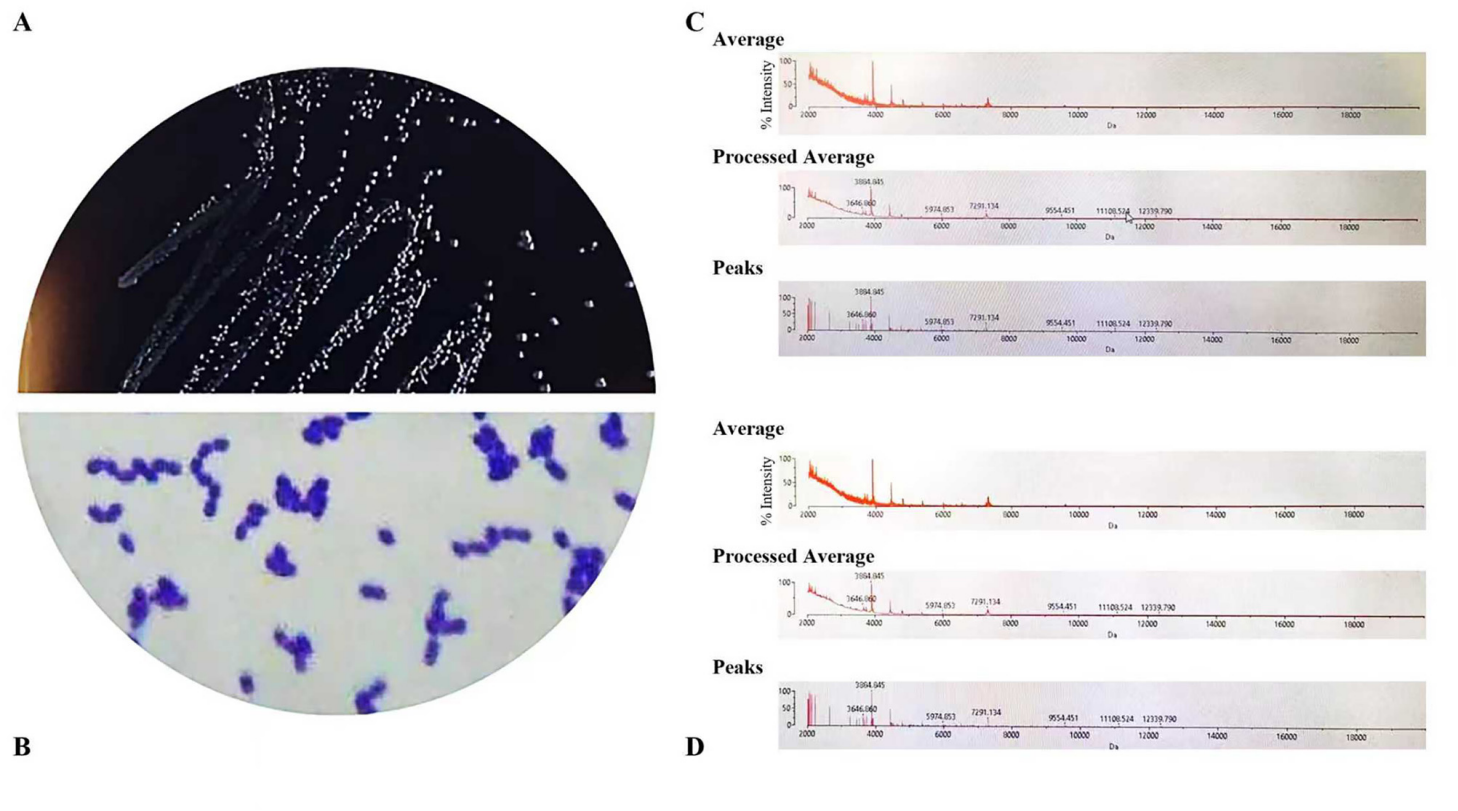
Isolation and identification of *E. faecalis* isolates from fresh feces of the observational and healthy control groups. 1a: Bacterial colony grew on the Pfizer Enterococcus selective Agar. 1b: Smear with Gram stain and under micoscopy. 1c: Spectrum on *E. faecalis identified by* MALDI-TOF mass spectrometer. 1d: Spectrum on *E. faecalis identified by* MALDI-TOF mass spectrometer. *E. faecalis, Enterococcus faecalis*.

The bacteria were identified by morphology and biochemical reaction along with the V-K 2 Compact Identification Card and V-K MALDI-TOF mass spectrometer, consistent with the characteristics of *E. faecalis*. A total of 81 strains were isolated from fresh feces of patients with CRC and healthy controls and identified as *E. faecalis,* respectively (Figure 1a–d).

### 3.3 Extraction of genome

**Figure 2:**
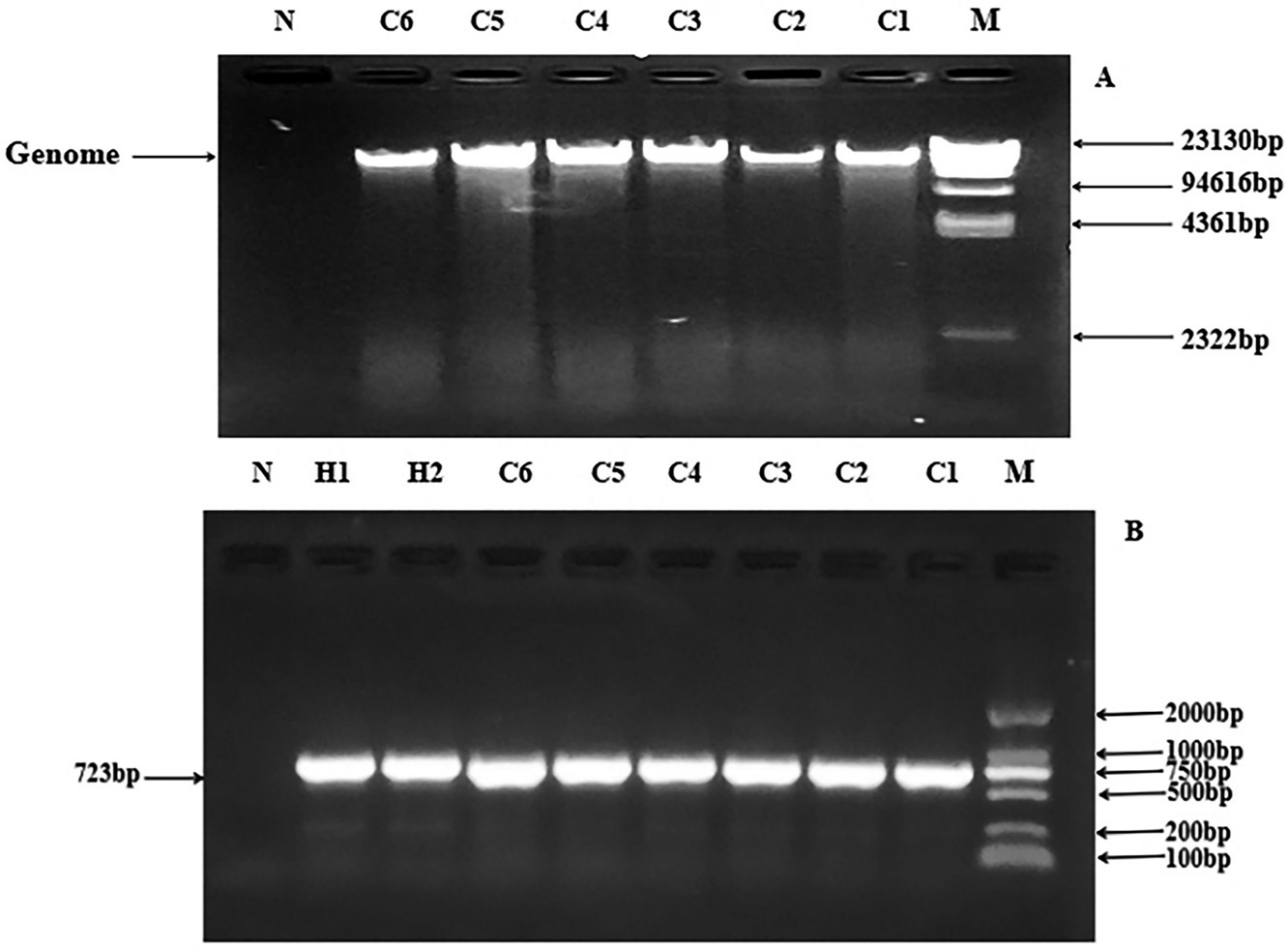
Agrose electrophoresis of bacterial genome extracted from *E. faecalis* isolates and amplicons of *NfsA* gene amplified by PCR. (a) Agrose electrophoresis of bacterial genome extracted from *E. faecalis* isolates. (b) Agrose electrophoresis of amplicons of *NfsA* gene amplified by PCR. *E. faecalis, Enterococcus faecalis*; NfsA, nitroreductase A.

The genome of the bacterial genome from patients with CRC and healthy controls was extracted using a reagent kit method, and a bright genomic band appeared at 23,000 bp (Figure 2a).

### 3.4 Amplification, cloning, and blasting ananlysis of *NfsA* gene

**Figure 3:**
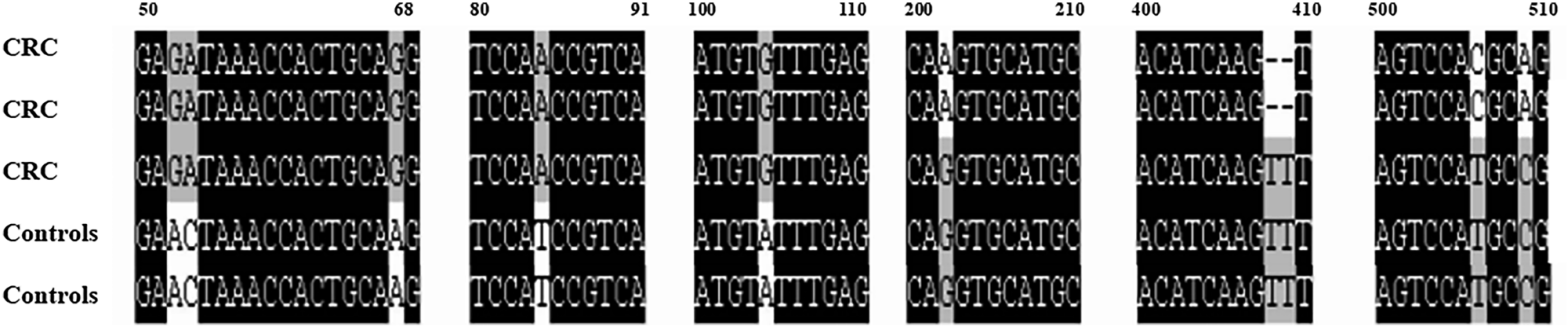
Multiple sequence alignment of *NfsA* gene in *E. faecalis* isolates using Clustal X (1.8). *E. faecalis, Enterococcus faecalis*; NfsA, nitroreductase A.

The *E. faecalis* genome of CRC patients and healthy controls showed bright target gene bands at 723 bp after PCR amplification and electrophoresis (Figure 2b). The recombinant plasmid was sequenced by Sangon Biotech (Shanghai, China) Co., Ltd. and multiple sequence alignment was conducted to compare mutation between the two groups. A total of 12 mutations were observed in the NfsA gene among *E. faecalis* isolates from CRC patients and healthy controls. The exact point mutation exhibited that the base mutated from GA to AC at 52 and 53rd, G to A at 67th, G to A at 105th, C to T at 506th, A to C at 509th in addition to a base deletation at 408, 409th. The mutations frequencies showed statistically significant (*P* < 0.05), as shown in Table 2 and Figure 3.

**Table 2:** Comparison of mutation frequency of sequences in *NfsA* genes [*n* (*η*/%)].

Groups	GA→AC at 52, 53^th^	G→A at 67^th^	G→A at 105^th^	Base deletion at 408, 409^th^	C→T at 506^th^	A→C at 509^th^
CRCs (*n* = 51)	36 (70.59)	29 (56.86)	20 (39.22)	18 (35.29)	17 (33.33)	18 (35.29)^a^
Controls (*n* = 30)	7 (23.33)	5 (16.67)	4 (13.33)	4 (13.33)	4 (13.33)	4 (13.33)

^a^ *P* < 0.05 versus control groups.

### 3.5 Extraction of total bacterial RNA and amplification of an internal reference gene

**Figure 4:**
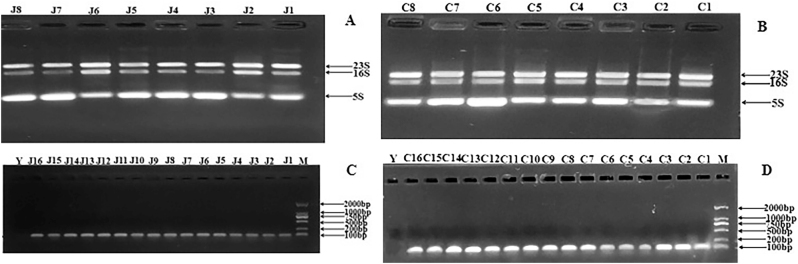
Agrose electrophoresis of total bacterial RNA and amplification of an internal reference gene. (a) Agrose electrophoresis of total bacterial RNA extracted from *E. faecalis* isolates from healthy controls. Lane J1-J8 represented the healthy controls. *E. faecalis, Enterococcus faecalis;* (b) Agrose electrophoresis of total bacterial RNA extracted from *E. faecalis* isolates from CRC patients. Lane C1-C8 represented the CRC patients. *E. faecalis: Enterococcus faecalis*; CRC, colorectal cancer. (c) Agrose electrophoresis of amplification of an internal reference gene from *E. faecalis* isolates from healthy controls. Lane J1-J16 represented the healthy controls. Y: negative control. M: marker. *E. faecalis, Enterococcus faecalis.* (d) Agrose electrophoresis of amplification of an internal reference gene from CRC patients. Lane C1-C16 represented the CRC patients. Y: negative control. M: marker. *E. faecalis, Enterococcus faecalis*; CRC, colorectal cancer.

Total bacterial RNA including 23s, 16s, and 5s RNA was successfully extracted from the genome of *E. faecalis* in the observational and healthy control group, respectively (Figure 4a and b). The internal reference gene bands appeared at 109 bp after total RNA reverse transcription PCR amplification and electrophoresis in the observational and healthy control group (Figure 4c and d).

### 3.6 Fluorescence quantitative PCR of *NfsA* cDNA between two groups

**Figure 5:**
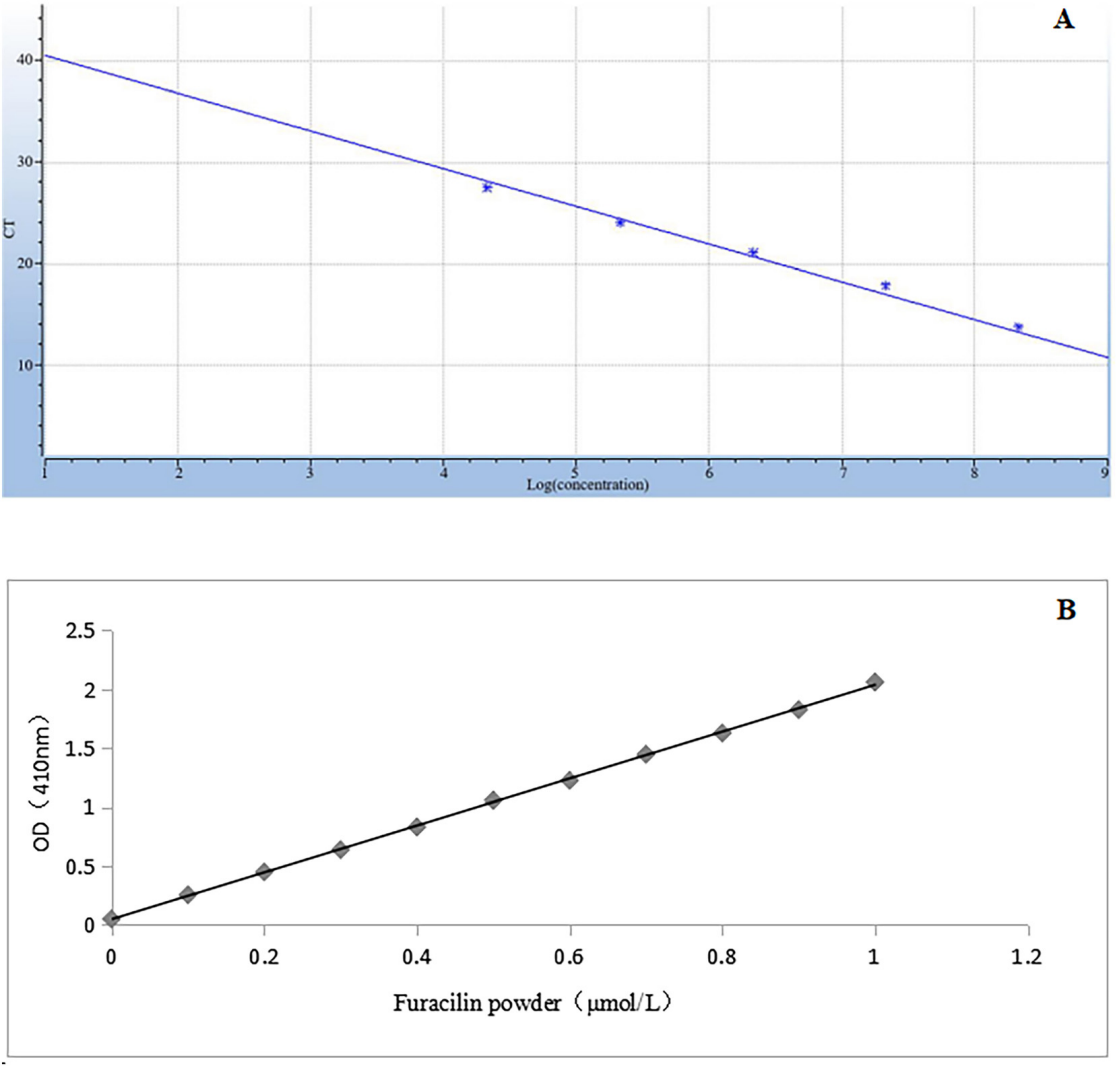
Standard curves used for calculation of *NfsA* mRNA and activity. (a) The standard curve automatically generated by the system according to *C*_*t*_ value and copy number of the internal reference gene used for calculation of *NfsA* mRNA. (b) The standard curve for furacilin powder was calculated for the *NfsA* activity. NfsA, nitroreductase gene A.

The standard curve was automatically generated by the system according to *C*_*t*_ value and copy number with: *y* = −3.742 *C*_*t*_ + 44.353 (coefficient of determination *R* ^2^ = 0.996, slope *k* = −3.742, and intercept *b* = 44.353) (Figure 5a). The expression of NfsA mRNA in the observational group was higher than that of healthy control group, and the difference was statistically significant (*P* < 0.05), as shown in Table 3.

**Table 3:** Results of *NFsA* gene expression mRNA contents (*X ± s*, pg/μL).

Groups	*NfsA* mRNA
Observational group	2.16 ± 1.16^a^
Control groups	1.08 ± 1.12
*F*	0.415
*P*	0.021

^a^ *P* < 0.05 versus control groups. *NfsA*, nitroreductase gene A.

### 3.7 Analysis of the NfsA activity

The standard curve: *y* = 1.9866 *x* + 0.0493 resulted in a coefficient of determination *R* ^2^ of 0.9996 (Figure 5b). Compared with the healthy control group, the NfsA activity in the observational group was significantly increased and exhibited a statistically significant difference (*P* < 0.05), as are shown in Table 4.

**Table 4:** Results of *NFsA* expression activity (*X ± s*, U/L).

Groups	NfsA activity
Observational group	46.73 ± 5.02^a^
Control groups	40.23 ± 4.46
*F*	0.084
*P*	<0.05

^a^ *P* < 0.05 versus control groups. *NfsA*, nitroreductase gene A; CRC, colorectal cancer.

## 4 Discussion

In this study, we confirmed the NfsA sequence, mean copies of mRNA expression, and activity among *E. faecalis* isolates in people with CRC in comparison with healthy individuals in order to investigate the association of this species with CRC.

The multiple sequence alignment results revealed that a total of 12 mutations were observed in NfsA sequences and the mutations frequencies showed statistically significant between the two groups. In meanwhile, the mean copies of NfsA mRNA expression and activity also showed statistically significant between the two groups.

Some studies on fecal microbial flora and gastrointestinal cancers, especially CRC, have been reported, indicating a difference in fecal microbial flora among people with CRC and healthy individuals [3], 17]. Our previous study reported that the structure of microflora in CRC patients is different from that in healthy people in China [18]. CRC can be associated with host microbiome dysbiosis [19], 20]. Although the most prevalent strain found in human feces is *E. faecalis* (10^5^–10^7^ colony-forming units (CFU)/g) among the enterococci that colonize the GI tract, these numbers can change with the host’s geographical location, and especially, diet [21]. *E. faecalis* can grow uncontrolled, thus increasing the possibility of new mutations that can modify its virulence and also change the final product of its metabolism, becoming potentially harmful to the epithelial tissue. This could be an explanation for the increased concentrations of *E. faecalis* that were found in some studies on CRC patients’ feces, as well as its harmful role in immunodeficient mice [22]. *E. faecalis* has been introduced as a causative agent for oxidative stress [12], 23]. *E. faecalis* damage the colon epithelial DNA by producing reactive oxygen radicals. Additonally, the accumulated studies have found that nitrite is further combined with nitrogen-containing compounds to produce nitroso compounds, which are highly carcinogenic [24]. More studies also indicated that bacterial metabolites secondary such as deoxycholic acid can produce reactive oxygen free radicals, which can cause DNA breaks in intestinal mucosal cells and chromosomal instability to induce tumorigenesis [16], 25].

In the present study, we found that NfsA mRNA expression and NfsA activity higher in the observational group than the control group. This demonstrated that NfsA excreated from *E. faecalis* isolates could reduce nitrate in the human gastrointestinal tract to active and toxic nitrite. Nitroso compounds play a role in the formation of adenomatous polyps and CRC [26]. Nitrogen-containing compounds is a secondary metabolite of *E. faecalis* that can interfere with the cell cycle. This disorder may triggers the development and extension of CRC [27]. Notably, some confounding factors including age, cigarete, fat diet were evaluated. There was a difference in the high fat diet of subjects in the observational group. This result is consist with previous reporters [28]. Evidence from this findings suggests the intervention of *E. faecalis* in inducing CRC. Among them, dietary intervention is one of the most commonly used methods with high feasibility and safety. Animal experiments found that high-fat diet caused excessive expression of adipokines and cytokines in mice to increase the risk of CRC, and excessive intake of red meat and carcinogenic substances in the intestine also increased the risk of CRC in normal people [27], 29]. Practically, intake of plant foods such as fiber-diet and cereals can prevent the incidence of CRC, which may be related to the production of anticancer substances such as fatty acids by bacterial fermentation of dietary fiber [26], 30]. On the other hand, use of supplemental probiotics and fecal microbiota transplantation is also the focus of many key studies. Butanol-producing bacteria, such as butyrobacter and *Bacillus subtilis*, inhibit the development of CRC induced by DMH in mouse models, which involves reducing inflammation and maintaining immune homeostasis [31]. Another probiotic *Lactobacillus casei* (strain BL23) not only successfully inhibits the development of CRC in the mouse model, but also regulates the steady-state development of the intestinal flora to a healthy state [32], 33].

However, the sample size of CRC patients was small, and there was no obvious staging of CRC patients. Therefore, the sample size should be increased in the future, and TNM staging should be performed on malignant tumors in patients with intestinal cancer the further to improve the study.

## 5 Conclusions

In summary, the NfsA sequence, mRNA expression, and activity of NfsA in fecal samples of patients with CRC were found to differ from those of healthy controls. *E. faecalis* colonizing within intestine may play a role in the tumorigenesis. Therefore, it is necessary to monitor the *E. faecalis* changes in intestinal gut and their metabolites to predict the occurrence and development of CRC in order to achieve the effect of early prevention and treatment of CRC.
